# Reproductive compatibility among populations and host‐associated lineages of the common bed bug (*Cimex lectularius* L.)

**DOI:** 10.1002/ece3.6738

**Published:** 2020-10-07

**Authors:** Zachary C. DeVries, Richard G. Santangelo, Warren Booth, Christopher G. Lawrence, Ondřej Balvín, Tomáš Bartonička, Coby Schal

**Affiliations:** ^1^ Department of Entomology University of Kentucky Lexington KY USA; ^2^ Department of Entomology and Plant Pathology North Carolina State University Raleigh NC USA; ^3^ Department of Biological Science The University of Tulsa Tulsa OK USA; ^4^ Ecology and Evolutionary Biology Princeton University Princeton NJ USA; ^5^ Department of Ecology Faculty of Environmental Sciences Czech University of Life Sciences Prague Prague Czech Republic; ^6^ Department of Botany and Zoology Masaryk University Brno Czech Republic; ^7^ W.M. Keck Center for Behavioral Biology North Carolina State University Raleigh NC USA; ^8^ Center for Human Health and the Environment North Carolina State University Raleigh NC USA

**Keywords:** *Cimexlectularius*, host‐associated differentiation, reproduction, speciation, *Wolbachia*

## Abstract

As populations differentiate across geographic or host‐association barriers, interpopulation fertility is often a measure of the extent of incipient speciation. The bed bug, *Cimex lectularius* L., was recently found to form two host‐associated lineages within Europe: one found with humans (human‐associated, HA) and the other found with bats (bat‐associated, BA). No unequivocal evidence of contemporary gene flow between these lineages has been found; however, it is unclear whether this is due to an inability to produce viable “hybrid” offspring. To address this question and determine the extent of compatibility between host‐associated lineages, we set up mating crosses among populations of bed bugs based on both their host association (human—HA vs. bat—BA) and geographic origin (North America vs. Europe). Within‐population fecundity was significantly higher for all HA populations (>1.7 eggs/day) than for BA populations (<1 egg/day). However, all within‐population crosses, regardless of host association, had >92% egg hatch rates. Contrary to previous reports, in all interlineage crosses, successful matings occurred, fertile eggs were oviposited, and the F_1_ “hybrid” generation was found to be reproductively viable. In addition, we evaluated interpopulation genetic variation in *Wolbachia* among host‐associated lineages. We did not find any clear patterns related to host association, nor did we observe a homogenization of *Wolbachia* lineages across populations that might explain a breakdown of reproductive incompatibility. These results indicate that while the HA and BA populations of *C. lectularius* represent genetically differentiated host‐associated lineages, possibly undergoing sympatric speciation, this is in its incipient stage as they remain reproductively compatible. Other behavioral, physiological, and/or ecological factors likely maintain host‐associated differentiation.

## Graphical abstract

The bed bug, *Cimex lectularius* L., was recently found to form two host‐associated lineages within Europe: one found with humans (human‐associated, HA) and the other found with bats (bat‐associated, BA). No unequivocal evidence of contemporary interlineage gene flow between these lineages has been found; however, it is unclear whether this is due to an inability to produce viable “hybrid” offspring. Our results show these lineages to be reproductively compatible, suggesting other behavioral, physiological, and/or ecological factors likely maintain host‐associated differentiation.

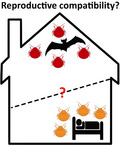

## 1 INTRODUCTION

Understanding the mechanisms responsible for incipient speciation is critical to our understanding of evolution. Recently, the bed bug, *Cimex lectularius*, an ectoparasite frequently associated with humans (Usinger, 1966), was discovered to have two morphologically and genetically differentiated lineages: one associated with humans (HA = human‐associated) and another associated with bats (BA = bat‐associated) (Balvín, Munclinger, Kratochvíl, & Vilímová, 2012). Mitochondrial data suggest that these lineages diverged ~245,000 years ago (95% confidence interval 98,696–866,522 years ago) (Balvín et al., 2012). Despite strong geographic overlap, there is no unequivocal evidence of contemporary gene flow between these lineages, as assessed with both mitochondrial and nuclear (microsatellite loci and variation in insecticide resistance gene sequences) markers (Booth, Balvín, Vargo, Vilímová, & Schal, 2015). Broad geographic overlap of *C. lectularius* populations and the lack of interlineage gene flow suggest that these host‐associated lineages have differentiated into two host races that may be undergoing incipient, and arguably sympatric, speciation.

Differentiation of host races has been extensively studied in phytophagous insect specialists. Perhaps the best understood host‐race system is that of the apple maggot fly, *Rhagoletis pomonella* (Walsh). In *R. pomonella*, there is a clear divergence among host lineages, with some preferentially attracted to apples and others to hawthorn (Bush, 1969). These lineages can be distinguished genetically (Feder, Chilcote, & Bush, 1988; McPheron, Smith, & Berlocher, 1988), and their isolation appears to be reinforced by odor‐based discrimination between the alternate host plants, possibly driven by only a few genes (Dambroski et al., 2005; Linn et al., 2003).

Several mechanisms have been evaluated for restricting gene flow between the two host‐associated lineages of *C. lectularius*. Wawrocka and Bartonička (2013) reported lower fecundity and survivorship of all life stages when reared on non‐native host blood (e.g., human blood for BA bed bugs, bat blood for HA bed bugs) than on native host blood. Wawrocka, Balvín, and Bartonička (2015) further showed that HA and BA bed bugs were reproductively incompatible, with no eggs produced from interlineage crosses despite mating and sperm transfer. Complete reproductive incompatibility would support that these host races have evolved to at least two distinct biological species; however, with whole mitogenomic divergence of <2% (Booth, W., pers obs), these could not be considered highly diverged cryptic species (Hebert, Ratnasingham, & De Waard, 2003). Despite a lack of contemporary gene flow, behavioral isolating mechanisms remain elusive. *Cimex lectularius* use aggregation pheromone to orient to and arrest within bed bug‐conditioned shelters (Gries et al., 2015; Siljander, Gries, Khaskin, & Gries, 2008). Balvín, Bartonička, Pilařová, DeVries, and Schal (2017) showed that *C. lectularius* from the HA and BA lineages were incapable of discriminating lineage‐specific shelters, and thus might co‐aggregate in situations where both lineages might overlap in the wild. We recently extended these observations, demonstrating that both HA and BA *C. lectularius* could not discriminate shelters conditioned by a congeneric bat bug, *Cimex pipistrelli* Jenyns (DeVries, Mick, Balvín, & Schal, 2017). Preliminary studies also revealed that interlineage *C. lectularius* crosses produced viable offspring (DeVries et al., 2017), suggesting that disparate results on reproductive compatibility may relate to technical and methodological differences between studies.

In addition, *C. lectularius* harbors *Wolbachia*, a Gram (−), intracellular *α*‐proteobacterium, as an obligate nutritional mutualist that provisions the bed bug with riboflavin and biotin (Hosokawa, Koga, Kikuchi, Meng, & Fukatsu, 2010). *Wolbachia* has been shown to drive host reproductive phenotypes, including cytoplasmic incompatibility and male‐killing in various species (Werren, 1997). Experimental crosses of *C. lectularius* males with *Cimex columbarius* Jeyns females (closely related and sometimes considered a subspecies) resulted in fewer eggs oviposited than in the reciprocal cross (Ueshima, 1964), resembling a cytoplasmic incompatibility‐like pattern. If reproductive incompatibilities among bed bug populations could be explained by lineage‐specific *Wolbachia* variants, potentially explaining the results of Wawrocka et al. (2015), then a lack of variation among assayed populations, or a homogenization of *Wolbachia* variants due to cross‐contamination through a communal feeding system, could theoretically remove such incompatibility.

To better understand reproductive compatibility between different host‐associated lineages of *C. lectularius*, we (a) investigated the relationship between assayed populations using mitochondrial markers previously found to reveal host‐lineage differentiation; (b) conducted reproductive crosses that spanned geographic locations, and both within and between the two host‐associated lineages; and (c) investigated the patterns of genetic variation of *Wolbachia* among populations for evidence of interpopulation homogenization.

## 2 MATERIALS AND METHODS

### 2.1 Experimental animals

Six populations of *C. lectularius* were used in this study. Collections were made from bat roosts and homes, and from both the United States and Europe. A full description of each population is provided in Table 1. It should be noted that bat‐associated populations were only collected in the Czech Republic, and these lineages have not been documented outside of Europe. After collection, populations were maintained in the laboratory in plastic jars (6 cm diameter × 7 cm high) at 27°C and ~50% RH. All populations were fed defibrinated rabbit blood through an artificial feeding system that utilized a heated water bath (B. Braun Biotech Inc.) to circulate water at 37°C through custom‐designed water‐jacketed glass feeders. Blood was retained in the feeders by an artificial membrane (grafting tape; A.M. Leonard) through which bed bugs could feed on warmed blood. This feeding method was used for all experiments (as detailed below). Field‐collected bed bugs were reared in the laboratory through at least two generations prior to testing.

**TABLE 1:** Bed bug populations used in reproductive crosses

Population	Host	Location	Year collected
HA‐JC	Human	Jersey City, NJ, USA	2008
HA‐WS	Human	Winston Salem, NC, USA	2008
HA‐OS	Human	Oslo, Norway	2014
HA‐BE	Human	Beroun, Czech Republic	2015
BA‐MO	Bat (*Myotis myotis*)	Moravicany, Czech Republic	2014
BA‐HN	Bat (*Myotis myotis*)	Hanusovice, Czech Republic	2014

### 2.2 Molecular confirmation of *Cimex lectularius* host lineage

Five representative samples from each population were selected for mitochondrial DNA (mtDNA) sequencing in order to compare with patterns of host‐lineage association previously observed by Balvín et al. (2012) and Booth et al. (2015). Samples were sequenced for both a 559‐bp fragment of cytochrome oxidase subunit I (COI), using the LepF (5′‐ATT CAA CCA ATC ATA AAG ATA TNG G‐3′), LepR (5′‐TAW ACT TCW GGR TGT CCR AAR AAT CA‐3′) (modified from Hajibabaei, Janzen, Burns, Hallwachs, & Hebert, 2006), and a 338‐bp fragment of 16S rRNA using LR‐J‐13007 and LR‐N‐13398 primers (according to Szalanski, Austin, McKern, Steelman, & Gold, 2008). PCR protocols and bidirectional sequencing of PCR products followed those outlined in Balvín et al. (2012). Sequence alignments were performed using CLC Main Workbench v.7.6.2 (Qiagen, https://www.qiagenbioinformatics.com). As in Balvín et al. (2012) and Booth et al. (2015), genes were concatenated for further analysis (total 897 bp). A median‐joining network was constructed using TCS: Phylogenetic Network Estimation Using Statistical Parsimony software (Clement, Posada, & Crandall, 2000). Sequences were aligned to those presented in Booth et al. (2015).

### 2.3 Reproductive compatibility and F_1_ viability

Reproductive compatibility was assessed using the methods described by DeVries et al. (2017). Briefly, fifth‐instar nymphs were fed, isolated, and allowed to eclose to adults, thus ensuring that all adults used in the experiments had not previously mated. Adults were then combined into same‐sex groups by population and fed. After one week, a second feeding was performed and single male/female pairs were introduced into 7.5‐ml glass vials. These were allowed 6 d to freely mate and lay eggs. A paper insert within the vial served as shelter, oviposition substrate, and ramp to reach the feeder. After 6 days, adults were removed and the number of eggs was recorded in each vial. Eggs were monitored for the next 14 days, and the number of first instars was recorded. Finally, the offspring from each replicate cross were combined and reared under similar conditions as the founding colonies to assess their reproductive viability—the ability of the F_1_ generation to produce offspring. This measure was not quantitative, so viability is reported only as yes/no at the population level.

All assays were female‐centric, so each comparison was between females that mated with males from their own population and females that mated with males from a different population. Also, the large number of crosses would create substantial within‐population temporal variation. To minimize this variation, all crosses were run in a 2 × 2 matrix design. Thus, in a cross between populations A and B, females of population A were mated to males of populations A and B, and females of population B were mated to males of populations A and B. Although this design resulted in homogeneous crosses (e.g., female A mated to male A, female B mated to male B) being repeated, it ensured that each cross had concurrent within‐population positive controls (e.g., A females × A males), which were used to normalize all crosses involving that population (e.g., A in this example). All assays were conducted between May 2014 and April 2016.

### 2.4 Data analysis

Fecundity was compared among all within‐population crosses using ANOVA, with means compared using the Tukey–Kramer multiple comparison test. Egg hatch rate (percentage of eggs resulting in 1st instars) was compared among all within‐population crosses using the Kruskal–Wallis test. Fecundity was compared in crosses between populations using Student's *t* test because each 2 × 2 design included within‐population positive control crosses. Hatch rate in interpopulation crosses was assessed using the Kruskal–Wallis test. All analyses were performed in SAS 9.4 (SAS Institute).

### 2.5 Molecular characterization of *Wolbachia* among host lineages

Interpopulation genetic diversity of *Wolbachia* was assessed at three single nucleotide polymorphisms (SNPs). In a preliminary screening of a subset of samples collected across Europe (~100 infestations split evenly between BA and HA), these SNPs were found to be able to discriminate between BA and HA lineages; no U.S. samples have previously been screened to determine whether this pattern holds true (Booth, W, unpublished). PCR amplification was performed using the following primers: SNP‐1: forward—CGGTAATCCTTGGGTGCAAT, reverse—TCCAATAACGCTATCTGAAAGTCT; SNP‐4: forward—CCCTGTGTAATGGGAATTGG, reverse—GCAACTTCTACCACGGGATT; and SNP‐12: forward—GGGTTACAGTGGCCAGAATG, reverse—TGCTGATAAGCCACGTTTACC. PCRs were performed in 20 µl volumes each containing 1× PCR buffer, 2 mM MgCl_2_, 100 mM dNTPs, 2.5 mM of each primer, 0.5 U Taq DNA polymerase (Apex, Genesee Scientific), 50 ng of DNA template, and ddH_2_O to make 20 µl. PCR cycling conditions included an initial denaturation stage of 5 min at 95°C, followed by 35 cycles each consisting 1 min at 95°C, 1 min at 58°C, and 1 min at 72°C. This was followed by a final extension stage of 72°C for 5 min. PCR products were run on 2% 1× TBA agarose gels (with ethidium bromide) to confirm amplicons of the expected sizes (SNP‐1 = 227 bp, SNP‐4 = 239 bp, and SNP‐12 = 262 bp). PCR products were purified using ExoSAP‐IT (Affymetrix Inc.), bidirectionally sequenced using the BigDye Terminator v3.1 Cycle Sequencing Kit (Applied Biosystems), and run on an ABI 3130xl Genetic Analyzer (Applied Biosystems). Sequences were visualized and edited in CLC Main Workbench v.7.6.2 (Qiagen, https://www.qiagenbioinformatics.com).

## 3 RESULTS

### 3.1 Molecular confirmation of two host lineages

**FIGURE 1:**
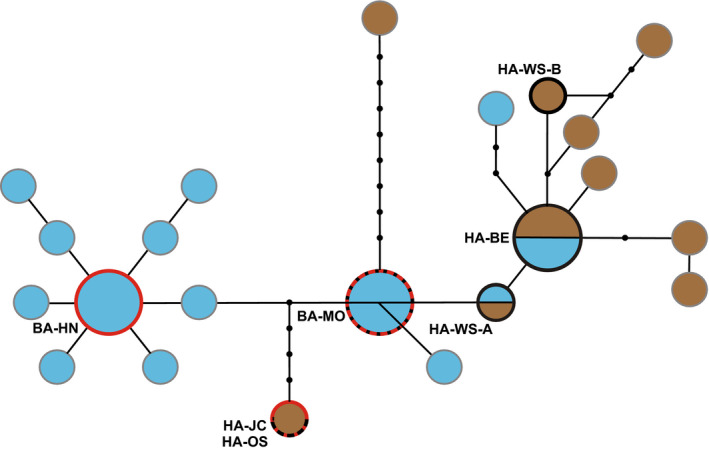
Haplotype network of human‐ and bat‐associated bed bug samples based on concatenated mitochondrial cytochrome oxidase subunit 1 and 16S rRNA gene sequences. Population names follow Table 1. Note that two haplotypes were detected in the WS population and were given the suffixes ‐A or ‐B. Haplotypes are derived from Booth et al. (2015). Blue circles indicate haplotypes found in bat‐associated samples, whereas brown indicate haplotypes found in human‐associated samples. Small black dots represent unsampled haplotypes. *Wolbachia* SNP variants for the populations tested here (labeled in red) are indicated by the haplotype circle outline. Gray = untested, red = bat‐associated, black = human‐associated, red/black hashed = both polymorphisms present at each locus. Note that while populations HA‐JC and HA‐OS shared the same human‐associated mtDNA haplotype, HA‐JC exhibited the bat‐associated SNP variants, while HA‐OS exhibited heterozygosity

Bat‐associated samples from the Czech Republic (Hanusovice [BA‐HN]: GenBank Accession #—COI—MT881768, 16S—MT882031; and Moravicany [BA‐MO]: GenBank Accession #—COI—MT881767, 16S—MT882034) were found to be identical to two BA haplotypes identified previously in Balvín et al. (2012) and Booth et al. (2015) [H3: GenBank Accession # GU985526.1, KJ937969; and H8: GenBank Accession # KJ937983, KJ937969, respectively, in Booth et al. (2015)]. Two haplotypes were found in the Winston Salem (USA) sample (HA‐WS‐A: GenBank Accession #—COI—MT881766, 16S—MT882029; and HA‐WS‐B: GenBank Accession #—COI—MT881766, 16S—MT882035, Figure 1), with the former previously found in both BA and HA samples in Europe (H25 in Booth et al., 2015; GenBank Accession # GU985525.1, KJ937977). Likewise, the Beroun (HA‐BE: Czech Republic: GenBank Accession #—COI—MT881765, 16S—MT882033) sample was found to exhibit a haplotype previously identified in both BA and HA samples (H2: GenBank Accession # GU985525.1, KJ937974). Jersey City (HA‐JC, USA: GenBank Accession #—COI—MT881770, 16S—MT882032) and Oslo (HA‐OS, Norway: GenBank Accession #—COI—MT881769, 16S—MT882030) samples possessed an identical haplotype (Figure 1). Winston Salem sample HA‐WS‐B possessed a unique haplotype not seen in previous studies, but nested within the HA cluster of the network.

### 3.2 Fecundity and hatch rate within populations

**FIGURE 2:**
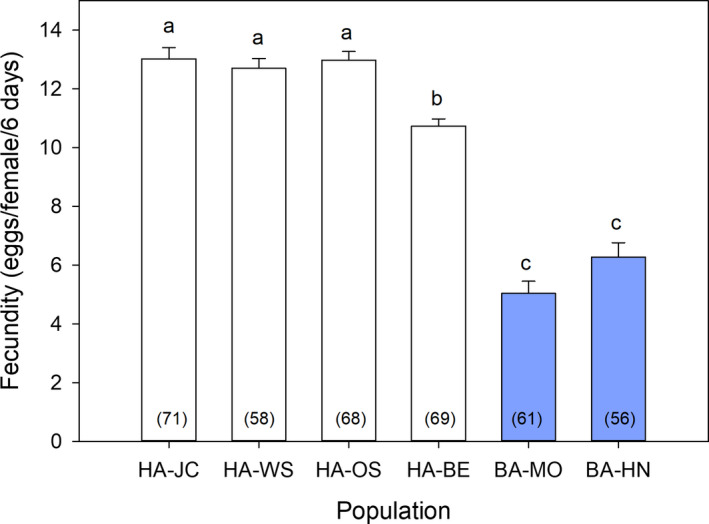
Average fecundity of bed bugs with different host associations (HA, human‐associated; BA, bat‐associated) in within‐population crosses (error bars represent *SEM*). Sample size is indicated in parentheses within each bar. Significant differences among populations based on ANOVA and Tukey's post hoc test (*p* < 0.05) are indicated with different lowercase letters

**FIGURE 3:**
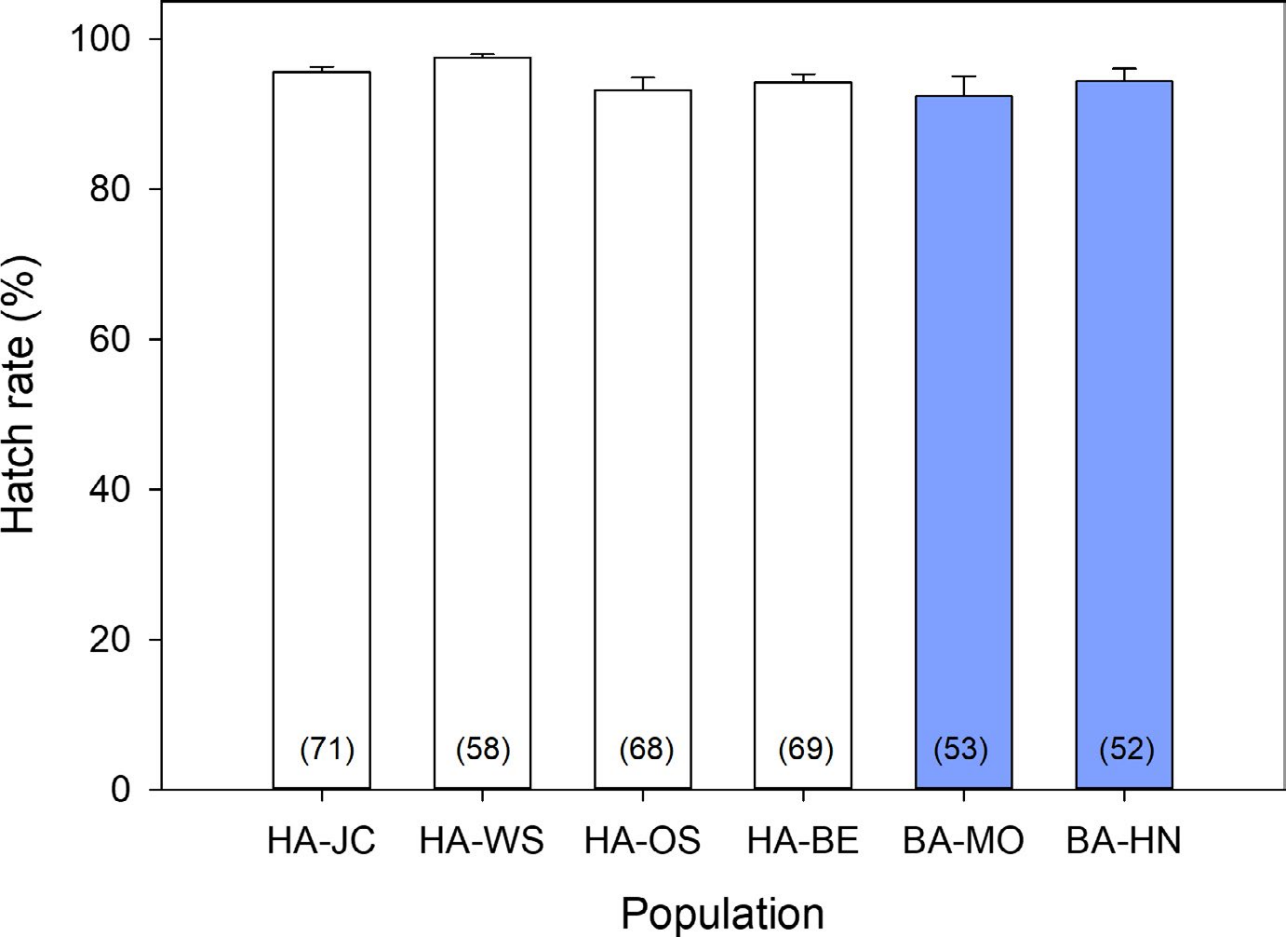
Average hatch rate of bed bugs with different host associations (HA, human‐associated; BA, bat‐associated) in within‐population crosses (error bars represent *SEM*). Sample size is indicated in parentheses within each bar. There were no significant differences among populations based on ANOVA (*p* = 0.1674)

Fecundity differed significantly among the populations tested (ANOVA, *F*_5,377_ = 96.17, *p* < 0.0001, Figure 2). Although there were some differences among the HA populations (HA‐BE significantly lower than the other three HA populations), fecundity in the four HA populations was significantly higher than in both BA populations. Hatch rate, however, was >92% in all six populations and was not significantly different among populations (Kruskal–Wallis test, *H*_5,365_ = 7.8037, *p* = 0.1674) (Figure 3).

### 3.3 Reproductive compatibility between populations

The interpopulation crosses revealed no evidence of reproductive incompatibility, with fecundity ranging from 70.7% to 147.9% of the respective within‐population fecundity (Table 2). Out of a total of 30 crosses between populations, only three resulted in significant changes in fecundity compared with the respective within‐population crosses. However, we could not detect any apparent patterns in these three crosses. Two crosses resulted in significant increases in fecundity: one involved HA populations (HA‐JC × HA‐WS) and one was between the two European host‐associated lineages (BA‐MO × HA‐OS). The single significant decline in fecundity was a within‐lineage cross between HA‐OS females and HA‐WS males (Table 2).

**TABLE 2:**
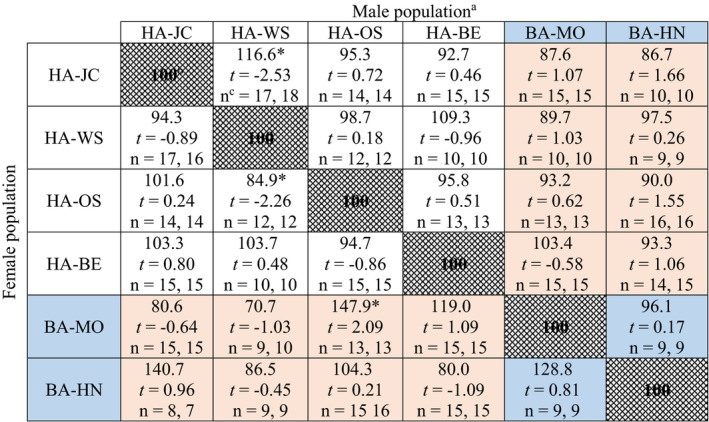
Relative fecundity in interpopulation crosses

^a^Human‐associated (HA) and bat‐associated (BA, shaded in blue) populations are shown.

^b^All percentages are relative to female fecundity in within‐population crosses (patterned cells set to 100%), and thus reflect the increase or decrease in fecundity when mated to males from another population.

^c^n represents the homogeneous sample size followed by the heterogenous sample size.

^*^Significant differences (*p* < 0.05) according to Student's *t* test (2‐tailed), with the test statistic and sample size (combined for the homogenous and heterogenous crosses) listed below each percentage (all tests with 1 *df*).

Hatch rate in the interpopulation crosses ranged from 72.4% to 111.1% of the respective within‐population hatch rates (Table 3). Only five out of the 30 interpopulation crosses resulted in significant changes in hatch rate, and notably, all five were interlineage crosses. All crosses between females from four HA populations and BA‐MO males produced eggs with significantly lower hatch rates (72.4%–83.5% of their respective within‐population hatch rate). However, hatch rates for the other BA population (BA‐HN) were not affected by intra‐ or interlineage mating, suggesting that lower hatch rate was unique to the BA‐MO population and thus represented a population effect and not a host‐associated lineage effect.

**TABLE 3:**
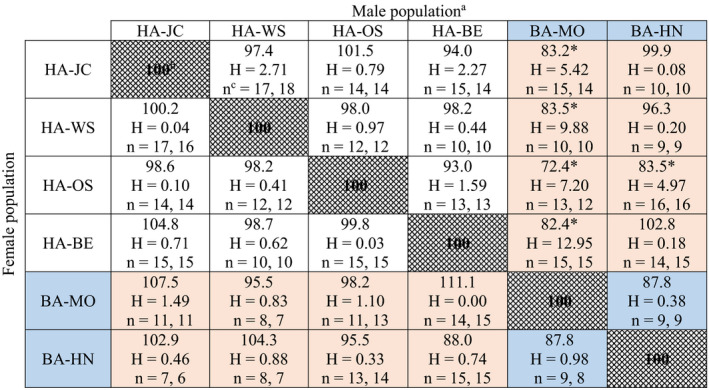
Relative hatch rate in interpopulation crosses

^a^Human‐associated (HA) and bat‐associated (BA, shaded in blue) populations are shown.

^b^All percentages are relative to hatch rate in within‐population crosses (patterned cells set to 100%), and thus reflect the increase or decrease in hatch rate when mated to males from another population.

^c^n represents the homogeneous sample size followed by the heterogenous sample size. Note: Only those crosses that produced eggs were used to calculate hatch rate. Therefore, sample size is lower than reported in Table 2 (fecundity) because some replicates did not produce any eggs.

^*^Significant differences (*p* < 0.05) according to the Kruskal–Wallis test, with the test statistic and sample size listed below each percentage (all tests with 1 *df*).

The F_1_ offspring of all interpopulation crosses (including interlineage crosses) were reared to adults, and examined for fertility. Combined progeny of interpopulation crosses produced offspring (F_2_ generation), indicating reproductive compatibility among all populations.

### 3.4 Molecular characterization of Wolbachia among host lineages

In order to assess whether interpopulation genetic variation exists in *Wolbachia*, and thus determine whether cross‐colony homogenization of *Wolbachia* lineages has occurred, three *Wolbachia* SNP variants were screened. Human‐associated samples from Beroun and Winston Salem exhibited *Wolbachia* SNP variants previously found to be associated with the human lineage in Europe (Booth, unpublished) (see haplotype circle outlines, Figure 1). Likewise, the BA sample from Hanusovice exhibited the expected BA *Wolbachia* SNP variants previously seen in the European bat lineage (Booth, W, unpublished). Interestingly, the U.S. HA‐JC sample exhibited *Wolbachia* SNP variants seen in the European bat‐associated lineages, and both the BA Moravicany, and the HA Oslo samples proved heterozygous for these SNPs (Figure 1).

## 4 DISCUSSION

The bed bug, *Cimex lectularius*, appears to be an excellent model for host‐associated genetic differentiation in sympatry. Its limited mobility (wingless adults), hematophagy, and close association of all life stages with the host make it a particularly attractive model for investigations of incipient speciation. Two host‐associated lineages co‐exist in Europe: human‐associated (HA) and bat‐associated (BA) populations (Balvín et al., 2012). These two lineages, or host races, have differentiated morphologically, behaviorally, and physiologically, suggesting adaptation to their respective hosts (Balvín et al., 2012; Wawrocka & Bartonička, 2013), and there is no unequivocal evidence of contemporary gene flow (Booth et al., 2015). Moreover, divergent insecticide selection pressures on HA and BA populations have led to resistance‐associated polymorphisms at insecticide target loci of HA, but not BA bugs (Balvín & Booth, 2018; Booth et al., 2015). Ultimately, lineage divergence based on host association is expected to promote host fidelity and reinforce further differentiation. Indeed, Wawrocka et al. (2015) showed compelling evidence that crosses between HA and BA bed bugs failed to produce any eggs, indicating reproductive incompatibility and the possible emergence of separate biological species.

Surprisingly, our results departed radically from those of Wawrocka et al. (2015). Crosses of all HA and BA pairs mated successfully, produced viable eggs that hatched, and yielded progeny that produced viable offspring. The differences in fecundity among populations appear to be inconsequential and not related to host differentiation. Interestingly, however, the two BA populations had significantly lower fecundity than the four HA populations in within‐population crosses. Although previous studies have documented variation in fecundity among HA populations (Barbarin, Barbu, Gebhardtsbauer, & Rajotte, 2014; Gordon, Potter, & Haynes, 2015), the almost twofold greater fecundity in HA than BA populations is striking. These differences, within our experiments, may be attributed to host blood type and differential adaptations to the laboratory conditions that are related to time in culture. Diet has been shown to affect fecundity, growth, and development in HA populations (Barbarin, Gebhardtsbauer, & Rajotte, 2013) and BA populations (Wawrocka & Bartonička, 2013). In our assays, however, all populations were reared on the same diet (defibrinated rabbit blood), so the observed differences likely represent physiological adaptations among the populations. The two U.S. HA populations we collected in 2008 (HA‐WS and HA‐JC) could be better adapted to laboratory conditions. Yet, the two European HA populations that were collected either concurrently, or after both BA populations were collected (HA‐OS and HA‐BE, respectively), had significantly greater fecundity than the BA populations. Overall, these observations suggest that time in the laboratory was not a key factor responsible for interlineage differences in fecundity. Although there were differences in fecundity among populations, all the eggs produced (regardless of population) had a high probability of hatching (>92%). While not directly tested in the current manuscript, high levels of egg survivorship suggest that *C. lectularius* can adapt to non‐native diets within only a few generations.

The differences between our present and previous preliminary findings (DeVries et al., 2017) and those of Wawrocka et al. (2015) could be due to a number of other factors. The specimens tested by Wawrocka et al. (2015) were collected as nymphs, reared in the laboratory to adults, and used in crosses; they likely did not fully adapt to laboratory conditions. Although *C. lectularius* can develop on a range of hosts (Usinger, 1966), it is still unclear whether they require any time to acclimate from one host to another. Many phytophagous insects are incapable of switching diets later in life (Karowe, 1989; Scriber, 1979), so it is plausible that the switch of diets could negatively affect reproduction and reproductive compatibility. Furthermore, *C. lectularius* maintain a symbiotic relationship with intracellular *Wolbachia* (Hosokawa et al., 2010), and host‐associated strain variation has recently been found (Lawerence, 2018). While in some insects *Wolbachia* strain variation among populations has been shown to potentially result in reproductive incompatibility (Sharon et al., 2010; Stouthamer, Breeuwer, & Hurst, 1999; Werren, 1997; Werren, Baldo, & Clark, 2008), no such evidence exists for *Cimex* species. In fact, evidence presented here suggests that *Wolbachia* does not drive incompatibility among the *C. lectularius* populations. As variation was found in the *Wolbachia* genotypes across our tested populations, the results can also not be explained by a possible homogenization of the *Wolbachia* strains among populations, due to communal feeding and/or mass rearing in close proximity.

Similar levels of reproductive compatibility have been reported in other host races of other species. In the case of the apple maggot fly, *R. pomonella,* where two genetically distinguishable host races have been reported (Feder et al., 1988; McPheron et al., 1988), viable hybrids can be produced between the races, although they are often selected against (Linn et al., 2004). In a similar example, the pea aphid, *Acyrthosiphon pisum* Harris, is also capable of forming less‐fit but viable hybrids between two host races found associated with alfalfa and red clover (Via, Bouck, & Skillman, 2000). In sympatric speciation, the difference between host‐race formation and true speciation can be linked to an ability of crosses between host races to produce viable offspring. Our results support a model of host‐race (or lineage) formation, not true species formation, because all crosses produced eggs that hatched (F_1_ generation), and when reared to adults, produced offspring (F_2_ generation).

The factor(s) responsible for preventing gene flow between human‐ and bat‐associated lineages of *C. lectularius* remain unclear, but in the absence of reproductive incompatibility, ecological (e.g., sheltering sites, diet, host ecology) and behavioral (e.g., activity periods, host preference) factors likely play a role. Fidelity to lineage‐ or population‐based aggregations was dismissed as a behavioral isolating mechanism (Balvín et al., 2017; DeVries et al., 2017). However, a range of other behaviors have not been investigated, and differential host attraction is a primary candidate. Bed bugs are attracted to human odors (DeVries, Saveer, Mick, & Schal, 2019; Harraca, Ryne, Birgersson, & Ignell, 2012; Liu & Liu, 2015), but it is unknown how specialized their odor preferences are and whether host attraction differs in HA and BA bed bugs. Additionally, sperm storage and/or preference may vary between intra‐ and interlineage matings. Although this would be unlikely to cause reproductive isolation, it could substantially decrease the rate of hybridization, which would still allow for host races to be maintained as stable entities (Drès & Mallet, 2002). Further testing is needed to better understand this system and the proximate mechanisms responsible for preventing gene flow between lineages.

In conclusion, our results suggest that reproductive compatibility does not appear capable of preventing gene flow between host‐associated lineages of *C. lectularius*. Although there were some differences in fecundity among lineages, all human‐ and bat‐associated populations tested were fully compatible with each other under laboratory conditions. Future work should focus on ecological factors (diet, microbiome), chemosensory specialization (such as host preferences, odorant, and gustatory receptors), and morphological adaptations (e.g., ability to climb or hold on to the host when it disperses) which may maintain genetic isolation. The recently completed genome sequence of *C. lectularius*, and particularly its well‐annotated chemosensory genes (Benoit et al., 2016), should facilitate discovery in this area.

## CONFLICT OF INTEREST

The authors have declared that no competing interests exist.

## AUTHOR CONTRIBUTION

**Zachary Curran DeVries:** Conceptualization (equal); Formal analysis (equal); Investigation (equal); Methodology (equal); Writing‐original draft (equal); Writing‐review & editing (equal). **Richard G Santangelo:** Investigation (equal); Writing‐original draft (equal); Writing‐review & editing (equal). **Warren Booth:** Conceptualization (equal); Formal analysis (equal); Funding acquisition (equal); Investigation (equal); Methodology (equal); Resources (equal); Writing‐original draft (equal); Writing‐review & editing (equal). **Christopher Lawrence:** Investigation (equal); Writing‐original draft (equal); Writing‐review & editing (equal). **Ondřej Balvín:** Writing‐review & editing (equal). **Tomáš Bartonička:** Writing‐review & editing (equal). **Coby Schal:** Conceptualization (equal); Formal analysis (equal); Funding acquisition (equal); Methodology (equal); Resources (equal); Writing‐original draft (equal); Writing‐review & editing (equal).

## Supporting information

Data S1

## Data Availability

Reproductive compatibility data are available as supplementary information.
